# Utility of the GeneXpert Mycobacterium tuberculosis/Rifampin (MTB/RIF) Assay on Paraffin-Embedded Biopsy Tissue Samples for Detecting Tuberculosis: Comparison With Histopathology

**DOI:** 10.7759/cureus.12048

**Published:** 2020-12-13

**Authors:** Atif A Hashmi, Samreen Naz, Syed Rafay Yaqeen, Omer Ahmed, Syed Inayat Ali, Muhammad Irfan, Anwar Kamal, Naveen Faridi

**Affiliations:** 1 Pathology, Liaquat National Hospital and Medical College, Karachi, PAK; 2 Internal Medicine, Baqai Medical University, Karachi, PAK; 3 Internal Medicine, Liaquat National Hospital and Medical College, Karachi, PAK; 4 Anatomy, Baqai Medical University, Karachi, PAK; 5 Statistics, Liaquat National Hospital and Medical College, Karachi, PAK

**Keywords:** genexpert mycobacterium tuberculosis/rifampin (mtb/rif) assay, mycobacterium tuberculosis, polymerase chain reaction, chronic granulomatous inflammation, caseation necrosis

## Abstract

Introduction

The Xpert Mycobacterium tuberculosis/Rifampin (MTB/RIF) assay is a qualitative nested real-time polymerase chain reaction (PCR) performed on the GeneXpert instrument system. Although, the utility of this technique for detecting tuberculosis (TB) in sputum and pus samples is well established, however, the usefulness of GeneXpert on biopsy samples is still a matter of debate. Therefore, in this study, we evaluated the utility of GeneXpert for detecting MTB in biopsy specimens diagnosed with TB.

Methods

A retrospective observational study was conducted at the Department of Pathology, Liaquat National Hospital and Medical College. The data collection period was between January 2016 and December 2018 over a period of three years. Specimens included trucut/incisional biopsies and lymph node excisions. Cases with a favoured histopathological diagnosis of TB were included in the study. The Xpert MTB/RIF assay was performed on the samples obtained from paraffin-embedded biopsy tissue material, and comparison of histological features with Xpert MTB/RIF assay was performed.

Results

A total of 114 cases were included in the study. The mean age of the patients was 40.53±16.83 years, and 57.9% of patients were male. 68.4% of cases were extra-nodal with the lung being the most common extra-nodal site. On histopathological analysis, caseation necrosis, epithelioid granulomas and Langhan's giant cells were present in 64.9%, 70.2%, and 59.6% cases, respectively. On GeneXpert PCR assay, MTB was detected in 26.3% cases. A significant association of MTB detection on Xpert PCR assay was noted with the presence of necrosis on histopathology.

Conclusion

In our study, we noted that the MTB detection rate by GeneXpert assay on histopathologically diagnosed cases of TB was only 26.3%, and the detection rate was significantly increased in the presence of caseation necrosis on a biopsy tissue sample. Despite the low detection rate due to rapid turnover time, GeneXpert assay is an excellent adjunctive tool for detecting MTB in paraffin-embedded tissue samples.

## Introduction

Tuberculosis (TB) is a prevalent infectious disease in developing countries, like Pakistan. Due to poor quality government health-care services and lack of an effective national TB control program, the disease is still considered a significant cause of morbidity and mortality in Pakistan [1]. Conventional diagnostic tests for TB include TB smear and culture, while histopathology is still considered a cornerstone diagnostic test for the diagnosis of TB. However, Ziehl-Neelsen (ZN) staining is seldom positive in histopathology samples, and the diagnosis of TB relies on the presence of necrotizing chronic granulomatous inflammation after the exclusion of other possibilities, such as fungal infections. Although TB culture is a sensitive and specific method, however, it is time-consuming and obtaining samples for culture, especially from intra-abdominal sites is difficult, and the yield is low when the specimen is not composed of pus.

With the rapid advancement of molecular diagnostics, various molecular-based tests were introduced for detecting TB. The Xpert Mycobacterium tuberculosis/Rifampin (MTB/RIF) assay is a qualitative nested real-time polymerase chain reaction (PCR) performed on the GeneXpert instrument system [2]. The Xpert MTB/RIF assay was primarily intended for sputum samples, and various studies have documented the diagnostic reliability of Xpert MTB/RIF for sputum and pus samples [3,4]. Although, the utility of this technique for detecting MTB in sputum and pus samples is well established, however, the usefulness of GeneXpert on biopsy samples is still a matter of debate. Therefore, in this study, we evaluated the utility of GeneXpert for detecting MTB in biopsy specimens diagnosed with TB.

## Materials and methods

A retrospective observational study was conducted at the Department of Pathology, Liaquat National Hospital and Medical College. The data collection period was between January 2016 and December 2018, over three years. Specimens included trucut/incisional biopsies and lymph node excisions. Cases with a favoured histopathological diagnosis of TB were included in the study. Histological features suggestive of TB included well-formed granulomas, Langhans giant cells and caseous necrosis. Also, special stains (periodic acid Schiff and silver stains) were performed to exclude fungal organisms. Cases with differentials that included other diagnoses, such as sarcoidosis, cat-scratch disease, toxoplasmosis, were excluded from the study. The Xpert MTB/RIF assay was performed on the sample obtained from paraffin-embedded biopsy tissue material, and comparison of histological features with Xpert MTB/RIF assay was performed.

Data analysis was performed using Statistical Package for Social Sciences (Version 26.0, IBM Inc., Armonk, USA). Chi-square, Fisher exact and Independent t-tests were used to check the association. The odds ratio was calculated using univariate binary logistic regression. p-values ≤ 0.05 were considered as significant.

## Results

A total of 114 cases were included in the study. The mean age of the patients was 40.53±16.83 years, and 57.9% of patients were males. 68.4% of cases were extra-nodal with the lung being the most common extra-nodal site. On histopathological analysis, caseation necrosis, epithelioid granulomas and Langhan's giant cells were present in 64.9%, 70.2%, and 59.6% cases, respectively. On GeneXpert PCR assay, MTB was detected in 26.3% cases. A significant association of MTB detection on Xpert assay was noted with the presence of necrosis on histopathology. Compared with other sites, the MTB detection rate was significantly higher in lung and cervical lymph node biopsy specimens. No significant association of MTB detection was noted with the presence of Langhan's type of giant cells and epithelioid granulomas (Table 1).

**Table 1:** Clinicopathological features of the population under study MTB/RIF: Mycobacterium tuberculosis/Rifampin, PCR: polymerase chain reaction, MTB: Mycobacterium tuberculosis *Chi-square test was applied **Mean±SD (standard deviation), independent t-test was applied ***Fisher Exact test was applied ****p-value significant as <0.05

Clinicopathological feature	Frequency (%)			p-value
	GeneXpert MTB/RIF PCR assay			
	MTB detected (n = 30)	MTB not detected (n = 84)	Total (n = 114)	
Gender*				
Male	16(53.3)	50(59.5)	66(57.9)	0.556
Female	14(46.7)	34(40.5)	48(42.1)	
Age (years)**	38.0±16.76	41.43±16.86	40.53±16.83	0.340
Age groups*				
≤25 years	4(13.3)	18(21.4)	22(19.3)	0.083
26–50 years	22(73.3)	42(50)	64(56.1)	
>50 years	4(13.3)	24(28.6)	28(24.6)	
Site*				
Nodal	12(40)	24(28.6)	36(31.6)	0.248
Extra–nodal	18(60)	60(71.4)	78(68.4)	
Individual sites***				
Lung	16(53.3)	30(35.7)	46(40.4)	0.028
Cervical LN	12(40)	12(14.3)	24(21.1)	
Abdominal LN	0(0)	10(11.9)	10(8.8)	
Omental nodule	0(0)	6(7.1)	10(8.8)	
Spine (bone)	2(6.7)	2(2.4)	8(7)	
Liver	0(0)	2(2.4)	2(1.8)	
Finger nodule	0(0)	2(2.4)	2(1.8)	
Wrist nodule	0(0)	2(2.4)	2(1.8)	
Appendix	0(0)	2(2.4)	2(1.8)	
Chest wall nodule	0(0)	2(2.4)	2(1.8)	
Axillary LN	0(0)	2(2.4)	2(1.8)	
Knee joint	0(0)	4(4.8)	4(3.5)	
Necrosis*				
Present	28(93.3)	46(54.8)	74(64.9)	<0.0001****
Absent	2(6.7)	38(45.2)	40(35.1)	
Granulomas*				
Present	18(60)	62(73.8)	80(70.2)	0.156
Absent	12(40)	22(26.2)	34(29.8)	
Langhan’s giant cells*				
Present	12(40)	34(40.5)	68(59.6)	0.964
Absent	18(60)	50(59.5)	46(40.4)	

Similarly, on binary logistic analysis, a significant association of MTB detection on Xpert assay was noted with the presence of necrosis on histopathology (Table 2).

**Table 2:** Odds ratio by univariate binary logistic regression for MTB detection on GeneXpert (MTB/RIF) PCR assay MTB: Mycobacterium tuberculosis, MTB/RIF: Mycobacterium tuberculosis/Rifampin, PCR: polymerase chain reaction, CI: confidence interval *Reference group **Significant as <0.05

Clinicopathological characteristics	Odds ratio	95% CI	p-value
Gender			
Male	0.777	0.336–1.799	0.556
Female*	1		
Age groups			
≤25 years	1.333	0.293–6.064	0.710
26–50 years	3.143	0.968–10.202	0.057
>50 years*	1		
Site			
Nodal	1.667	0.698–3.980	0.250
Extra-nodal*	1		
Necrosis			
Present	11.565	2.587–51.703	0.001**
Absent*	1		
Granulomas			
Present	0.532	0.221–1.280	0.159
Absent*	1		
Langhan’s giant cells			
Present	1.020	0.436–2.388	0.964
Absent*	1		

## Discussion

In this study, we found that the MTB detection rate by GeneXpert PCR assay on histopathologically diagnosed cases of TB was only 26.3%, and significant association of MTB detection on Xpert assay was noted with the presence of caseation necrosis on biopsy sample on histopathology.

Mycobacteria Growth Indicator Tube (MGIT) and Lowenstein-Jensen (L-J) medium based TB cultures are considered the gold standard for MTB detection. However, the intended samples are sputum or pus specimens that are not possible, especially in cases of intra-abdominal and extra-pulmonary TB. Additionally, culture takes a long time. To overcome these problems, various rapid PCR-based assays were introduced, and their applicability to different sample types was assessed. The reported sensitivity of these GeneXpert assays ranges from 80%-90% for pulmonary samples, and 50%-70% for extra-pulmonary samples, respectively [5]. Another study reported sensitivities of Xpert TB assay for bronchial brushing and biopsies to be 57.4% and 63.9%, respectively [6].

The main advantage of Xpert PCR assay for MTB is rapid turnover time. However, Xpert MTB PCR is still considered complimentary to MTB culture for detecting TB. Alternatively, its applicability for extra-pulmonary and nodal TB is still debatable. In our study, we noted the detection rate of 26.3% for biopsy specimens diagnosed with TB on routine histopathology. A study including 714 cases of pulmonary and extra-pulmonary TB samples concluded that the detection rates of MTB by GeneXpert assay were 23.85% and 18.41%, respectively, for pulmonary and extra-pulmonary samples [7].

A few studies have also evaluated the utility of GeneXpert assay for biopsy and cytology samples [8]. A study reported a sensitivity of 28.57% of GeneXpert assay compared with histopathology, for the diagnosis of abdominal TB. However, the sample size in this study was small (n = 21) [9]. We evaluated the detection rate of MTB by GeneXpert assay on biopsy specimens in a relatively larger sample size.

We acknowledge a few limitations of our study. First, TB culture or smear was not performed as all specimens were of biopsy samples. Second, the clinical and radiological information was not available as it was a laboratory-based study. Also, small sample size and retrospective study design are yet other limitations of the study.

## Conclusions

GeneXpert PCR assay is a rapid diagnostic tool for detecting MTB than conventional TB culture. However, the detection rate of MTB by Xpert PCR assay was only 26.3% in biopsy samples diagnosed as TB. Despite the low detection rate, the complementary role of Xpert PCR assay for MTB detection cannot be overlooked as obtaining culture sample for nodal and extra-pulmonary TB is not possible, in the absence of frank collection of pus. Furthermore, we noted that the detection date of MTB by Xpert PCR assay was significantly raised in the presence of caseation necrosis in the biopsy sample.

## References

[1] Khan AH (2017). Tuberculosis control in Sindh, Pakistan: critical analysis of its implementation. J Infect Public Health.

[2] Shao Y, Peng H, Chen C (2017). Evaluation of GeneXpert MTB/RIF for detection of pulmonary tuberculosis at peripheral tuberculosis clinics. Microb Pathog.

[3] Shi J, Dong W, Ma Y (2018). Genexpert mtb/rif outperforms mycobacterial culture in detecting mycobacterium tuberculosis from salivary sputum. Biomed Res Int.

[4] Abong J, Dalay V, Langley I (2019). Use of GeneXpert and the role of an expert panel in improving clinical diagnosis of smear-negative tuberculosis cases. PLoS One.

[5] Osei Sekyere J, Maphalala N, Malinga LA, Mbelle NM, Maningi NE (2019). A comparative evaluation of the new Genexpert MTB/RIF ultra and other rapid diagnostic assays for detecting tuberculosis in pulmonary and extra pulmonary specimens. Sci Rep.

[6] Zhang Q, Zhang Q, Sun BQ (2018). GeneXpert MTB/RIF for rapid diagnosis and rifampin resistance detection of endobronchial tuberculosis. Respirology.

[7] Mechal Y, Benaissa E, El Mrimar N (2019). Evaluation of GeneXpert MTB/RIF system performances in the diagnosis of extrapulmonary tuberculosis. BMC Infect Dis.

[8] Held M, Laubscher M, Workman L, Zar HJ, Dunn R (2017). Diagnostic accuracy of GeneXpert MTB/RIF in musculoskeletal tuberculosis: High sensitivity in tissue samples of HIV-infected and HIV-uninfected patients. S Afr Med J.

[9] Ahmad R, Changeez M, Khan JS (2018). Diagnostic accuracy of peritoneal fluid Genexpert in the diagnosis of intestinal tuberculosis, keeping histopathology as the gold standard. Cureus.

